# Elective surgery during a global health crisis – point incidence of mortality and complications in a Portuguese hospital: A cohort study

**DOI:** 10.1097/MD.0000000000045758

**Published:** 2025-11-07

**Authors:** Ana Lídia Rouxinol-Dias, Diana Rodrigues, Leonardo Ferreira, Patrícia Martins Lima, Patrícia Ramos, Cláudia Camila Dias, Joana Berger-Estilita, Cristina Granja

**Affiliations:** a Department of Anaesthesiology, Centro Hospitalar Universitário de São João EPE, Porto, Portugal; b RISE-Health, Centre for Health Technology and Services Research, Faculty of Medicine, University of Porto, Porto, Portugal; c Department of Community Medicine, Information and Health Decision Sciences (MEDCIDS), Faculty of Medicine of the University of Porto (FMUP), Porto, Portugal; d Knowledge Management Unit, Faculty of Medicine of the University of Porto (FMUP), Porto, Portugal; e Institute of Anesthesiology and Intensive Care, Salem Spital, Hirslanden Hospital Group, Bern, Switzerland; f Institute for Medical Education, University of Bern, Bern, Switzerland.

**Keywords:** COVID-19 pandemic, elective surgery, perioperative mortality rate (POMR), postoperative complications, SIGIC system

## Abstract

Elective surgeries are often restricted due to pandemics, resource shortages, natural disasters, and mass casualty events, which prioritize emergency care. During the coronavirus disease 2019 pandemic, elective surgeries were significantly affected, with a global estimate of over 20,00,000 cancellations/wk. This retrospective cohort study aims to determine the perioperative mortality rate (POMR) and major morbidity among elective surgical patients admitted to the postanesthetic care unit of a large Portuguese academic medical center during a pandemic wave, evaluating the influence of the national integrated surgical waiting list management system (SIGIC) on these outcomes. The study included 1642 patients admitted to the postanesthetic care unit at Centro Hospitalar Universitário de São João in October and November 2020. Primary outcome was POMR, defined as all-cause death within 30 days post-surgery. Secondary outcomes included postoperative complications, classified by the Clavien–Dindo system. Data were collected retrospectively and analyzed using logistic regression to identify predictors of mortality and major complications. The overall POMR was 0.7%. The 30-day postoperative complication rate was 24.2%, with major complications in 4.9% of patients. Higher ASA scores (III/IV), greater European Society of Anaesthesiology/European Society of Cardiology surgical risk, and plastic surgery were significantly associated with major complications, while weekend surgeries under the SIGIC system were linked to fewer complications. The study’s findings highlight that: older patients, males, and those with higher risk scores are more susceptible to major complications. The lower POMR compared to international rates may be due to the study’s specific focus on elective noncardiac surgeries. The unique operational adjustments during the coronavirus disease 2019 pandemic, including the use of SIGIC for weekend procedures, contributed to a reduction in major complications.

## 1. Introduction

Several situations can lead to restrictions in elective surgeries. Hurricanes, earthquakes and mass casualty events disrupt hospital operations, suspending or postponing elective surgeries to manage emergencies.^[1]^ Economic constraints and infection control measures within hospitals can also necessitate restricting elective surgeries to manage resources and prevent infections.^[1,2]^ Pandemics, such as coronavirus disease 2019 (COVID-19), where the patient surge led to insufficient staff or personal protective equipment, necessitated postponing elective procedures to prioritize patients with the disease and reduce transmission risks, often resulting in deferring nonurgent surgeries to maintain critical care capacity.^[3,4]^ The COVIDSurg Collaborative Group estimated a worldwide cancelation of >20,00,000 elective surgeries/wk.^[3,4]^

The pandemic highlighted several vulnerabilities within the healthcare system, including resource shortages, staff burnout, and lack of operating room capacity during regular hours.^[1,5]^ After the March 20th lockdown, Portuguese hospitals saw a 58% decrease in elective surgeries, similar to global reports.^[2,6,7]^ Despite governmental instructions to address the backlog, Portuguese hospitals only achieved a modest recovery, with a 21% decrease in elective surgeries during the inter-lockdown period from June to December 2020.^[2]^ This reflects resource strain and the reach of maximum response capacity.

One strategy to increase elective surgeries in Portugal, even during surge times like COVID-19, was implementing overtime work through the integrated management system of the waiting list for surgery (SIGIC), created in 2004 and implemented in 2005 by the Portuguese government.^[8]^ SIGIC aims to reduce surgery waiting times, ensure equitable access, promote system efficiency, and provide quality information and transparency.^[8,9]^

Patients are enrolled in a centralized surgical list managed by the hospital where registration occurs. The hospital may decide to perform the surgery as overtime work or transfer the patient to another unit that can respond promptly – performing the procedure in-house as additional production translates into incentives or payments for professionals based on production.^[8]^ Payment for each procedure is indexed to the all patients refined diagnosis related groups value (35–55% allocated to the team, the rest to the institution), considering conditions such as expected length of stay.

This system requires greater record accuracy, including biometric registration of the team and completion of surgical and safety checklists. To ensure greater efficiency, the team generally has a lower resident/specialist ratio than regular procedures.^[8]^ For many Portuguese hospitals, all weekend elective procedures (from Friday afternoon to Sunday) represent overtime work with financial rewards for professionals indexed to each procedure, integrated into SIGIC.

SIGIC is unique to the Portuguese healthcare system and stands out worldwide due to its integration of overtime work and financial incentives to manage elective surgery waiting times. Unlike many healthcare systems, where elective surgeries are postponed due to resource constraints, Portugal’s SIGIC allows hospitals the flexibility to perform additional procedures during off-hours, ensuring continuous surgical care. This system maximizes resource utilization, provides equitable access, and promotes overall system efficiency, setting it apart as an innovative model for managing surgical backlogs and improving patient care outcomes.

In this retrospective cohort study, we aimed to determine the perioperative mortality and major morbidity of elective surgical patients admitted to a large Portuguese academic medical center’s postanesthetic care unit (PACU) during a pandemic wave. This study also aim to analyze the role of the SIGIC system in shaping perioperative outcomes during the unprecedented challenges posed by the COVID-19 pandemic, examining its effectiveness in managing surgical demand and resource allocation under crisis conditions. This helps understand the pandemic’s effects on elective surgeries, highlighting healthcare system vulnerabilities and strengths. This information is vital for improving future crisis management strategies and ensuring preparedness for similar disruptions. Additionally, the insights gained can inform policy decisions, enhance patient safety, and lead to long-term improvements in surgical care practices and perioperative monitoring. Ultimately, the study provides benchmarks for perioperative outcomes, driving continuous improvements in surgical care practices.

## 2. Materials and methods

### 2.1. Ethics

The study adhered to the principles of the Declaration of Helsinki and received approval from the Ethics Committee of Centro Hospitalar Universitário de São João, Porto, Portugal (Approval No. 55-21; Chair: Prof Pedro Brito) on February 19, 2021. Due to the retrospective design and use of anonymized patient data, the committee waived the requirement for individual informed consent (Supplemental Digital Files 1 and 2, Supplemental Digital Content, https://links.lww.com/MD/Q617). Additionally, the study followed the strengthening the reporting of observational studies in epidemiology guidelines for observational studies.^[10]^

### 2.2. Study design and setting

We conducted a retrospective cohort study in the PACU of Centro Hospitalar Universitário de São João, a tertiary care hospital and large academic medical center in northern Portugal with 1100 beds and a workforce of 5500 people, including 1200 medical doctors.

### 2.3. Study outcomes

#### 2.3.1. Primary outcome

The primary outcome was the perioperative mortality rate (POMR), defined as all-cause death within 30 days following surgery divided by the total number of procedures.^[11]^ POMR and postoperative complication rates are essential indicators for assessing patient safety and the health quality performance of surgical institutions globally.^[12,13]^ The Lancet Commission on Global Surgery recommends POMR as one of the 6 key indicators for measuring the strength of a country’s surgical system.^[14]^ Beyond being a quality and safety indicator, perioperative morbimortality for elective surgeries is a relevant measure of health access by assessing the continuity and availability of health services during restrictive periods.^[15]^

#### 2.3.2. Secondary outcomes

Secondary outcomes included the severity of postoperative surgical complications, evaluated using the Clavien–Dindo classification.^[16]^ This system is a widely used method for grading the severity of surgical complications. It categorizes complications based on the type of intervention needed to address them. While some systems consider the nature of the complication itself, the Clavien–Dindo system emphasizes the kind of treatment required to fix the issue. The system has 5 grades, with I being the least severe and V being the most severe.^[16,17]^ Grade I includes minor deviations from the usual recovery process, requiring no additional interventions beyond medications like pain relievers or antibiotics; grade II includes complications requiring additional medications or interventions like blood transfusions; grade III includes complications needing surgical, endoscopic, or radiological procedures; grade IV refers to life-threatening complications requiring intensive care; and grade V represents death of the patient. The system allows surgeons to communicate complication severity clearly and consistently across different specialties and institutions. By categorizing complications, it helps assess a patient’s overall risk profile and potential need for additional care. The Clavien–Dindo system facilitates research on surgical outcomes and helps identify areas for improvement in surgical techniques and patient care.^[17,18]^ Similarly to GlobalSurg,^[19]^ we considered Clavien–Dindo grades III, IV, and V major complications.

We also assessed mortality within 60 days, time to death, postoperative length of hospital stay, the need for surgical reintervention and the impact of SIGIC on mortality and severity of complications.

### 2.4. Inclusion and exclusion criteria

All consecutive inpatients admitted to the PACU of Centro Hospitalar Universitário de São João who underwent elective surgical procedures between October and November 2020 were eligible to be included. Patients undergoing emergency or urgent surgeries, patients who underwent cardiac surgeries and those requiring specialized postoperative care in high-dependency units, such as neurosurgical patients, were excluded from the PACU analysis and admitted to their respective specialized ICUs. To ensure the accuracy and reliability of the study findings, any patient records with significant missing data necessary for the study analysis, such as missing postoperative complication details or mortality data, were also excluded.

### 2.5. Data collection

Five independent researchers (ALRD, DR, LF, PML, PR) collected data between April 3, 2021 and April 28, 2021 from patients admitted to the PACU between October and November 2020. The researchers extracted data from electronic medical records, surgeries’ electronic anesthesia and surgical reports and safety records, and included patient demographics, surgical details, anesthesia type, and postoperative outcomes. All study participants ‘ medical and nursing electronic records were also reviewed to evaluate the occurrence of postoperative complications identified during the in-hospital stay, or through subsequent events such as further admissions to emergency services and 30-day postoperative outpatient consultation when applicable. Data collection included random validation checks and consistency evaluations to ensure accuracy and reliability. Ethnic background and socioeconomic status weren’t routinely collected information and could not be included in the analysis.

### 2.6. Statistics

#### 2.6.1. Sample size

Sample size for mortality rate estimation was calculated according to an expected mortality rate of 1% (half the predicted for high income elective surgery in the GlobalSurg iniciative,^[20]^ lower than expected incidence of complications), a margin of error of 0.5%, confidence level of 95%, the required sample size would be approximately 1521 patients. This sample size would allow for a logistic regression with 7 to 35 independent variables, for a complication incidence around 30%, and rule of event/variable of 50 or 10, as proposed by Bujang et al or Peduzzi et al^[21,22]^ We anticipated that 2 months would be sufficient to achieve this number of elective surgical procedures. The sampling strategy of selecting October and November 2020 was intended to avoid the confounding effects of pandemic peaks and winter holidays while capturing a period indicative of pre-winter conditions that affect hospital resource availability.^[2,23]^ This approach ensured that the collected data accurately reflected the hospital’s operational and patient care reality during a critical but stable period.

#### 2.6.2. Statistical analysis

We described continuous and ordinal variables using medians and interquartile ranges (IQRs) and reported absolute and relative frequencies for categorical variables. For univariate analysis, we applied the Mann–Whitney *U* test to continuous variables and used the chi-square test or Fisher’s exact test for categorical variables, as appropriate. We performed all analyses pairwise, with a significance level set at 5%.

We reported missing data in each analysis, comparing the relative frequency for survival status and the presence or absence of major complications. Prior to any statistical analyses, including univariate comparisons, we analyzed a missing data distribution panel and applied multiple imputations to address missing data, incorporating pooled estimates from 10 imputations using a fully conditional specification imputation method with 10 iterations. This approach assumes that data are missing at random, meaning that the probability of missingness for a given variable depends only on observed variables, not on the value of the missing variable itself.

We performed logistic regression for predictors showing a marginal association with mortality and major complications in the univariate analysis (*P* < .2). We encoded nominal independent variables as dummy variables. We assessed the model’s accuracy using the area under the area under the curve and tested calibration with the Hosmer–Lemeshow goodness-of-fit test. We expressed the results of the multivariate model as β coefficients and adjusted odds ratios (OR) with 95% confidence intervals (CIs) and *P*-values. We conducted all data analyses using IBM Statistical Package for the Social Sciences Statistics for Windows, version 28 (IBM Corp., Armonk).

## 3. Results

**Figure 1.**
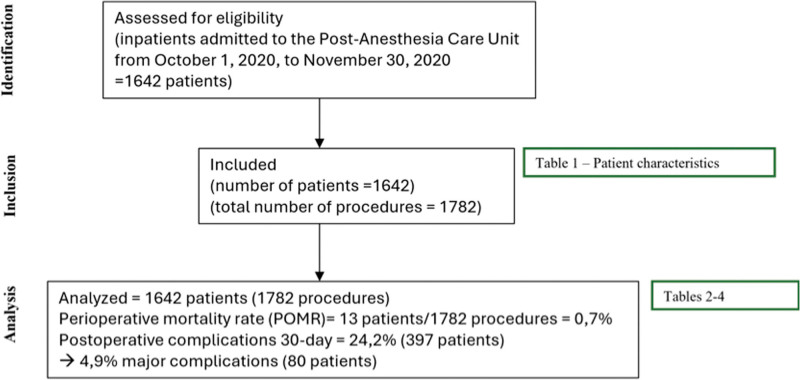
Study flowchart. POMR = perioperative mortality rate.

In October and November 2020, 1642 patients were admitted to the PACU, corresponding to 1782 postoperative admissions (Fig. 1). Table 1 describes the study population characteristics.

**Table 1:** Patient characteristics.

Characteristic	N = 1642 patients
Age (yr), median (IQR)	58 (IQR: 45–70)
Male, n (%)	781 (47.6)
ASA-PS, n (%; n = 1384)	
•I	227 (13.8)
•II	916 (55.8)
•III	457 (27.8)
•IV	42 (2.6)
Procedure duration (min; n = 1501)	Median: 105 (IQR: 65–165)
SIGIC, n (%)	574 (35.0)
Surgical specialty, n (%)	
•Gastrointestinal	132 (8.0)
•Hepatobiliary	125 (7.6)
•General – other subspecialties	309 (18.8)
•Orthopaedics	390 (23.8)
•Ear, nose, mouth, and throat	86 (5.2)
•Plastic	143 (8.7)
•Vascular	148 (9.0)
•Urology	221 (13.5)
•Others	88 (5.4)
ESAIC/ESC risk, n (%)	
•Low	778 (47.4)
•Intermediate	784 (47.7)
•High	80 (4.9)
Anaesthesia type, n (%; n = 1612)	
•General	804 (49.0)
•Locoregional	192 (11.7)
•Combined (general + locoregional)	613 (37.3)
•MAC	32 (1.9)

Ear, nose, mouth, and throat – includes maxillofacial, stomatology and otorhinolaryngology; SIGIC – overtime surgeries with additional fees per procedure for the team and the hospital; MAC. Only data from the first procedure during the study time was considered when a patient had multiple procedures. Missing data regarding ASA-PS for 258 (15.7%) patients, anesthesia type for 30 (1.8%) patients and procedure duration for 141 (8.6%) patients-multiple imputation aggregated results are presented for these variables.

ASA-PS = American Society of Anesthesiologists Physical Status, ESAIC = European Society of Anaesthesiology and Intensive Care, ESC = European Society of Cardiology, IQR = interquartile range, MAC = monitored anesthesia care, SIGIC = Sistema Integrado de Gestão de Inscritos para Cirurgia (Integrated Management System of the Waiting List for Surgery).

POMR: The overall postoperative mortality rate during the study period was 0.7% (13 patients died within 30 days after the procedure, within 1769 procedures).

Postoperative complications: The 30-day postoperative incidence of complications was 24.2% (397 patients), and 4.9% (80 patients) developed major complications, including 13 (0.8%) deaths within 30 days (Table 2).

**Table 2:** Description of complications according to Clavien–Dindo classification by procedure.

Complication Clavien–Dindo class	Cardiac	Respiratory (resp.)	Neurological (neuro.)	Gastrointestinal (GI)	Renal	Others	All
I, n (%)	7 (2.9)	5 (2.1)	22 (9.1)	25 (62.5)	7 (2.6)	177 (72.8)	243 (61.2)
II, n (%)	12 (16.7)	5 (6.9)	5 (6.9)	3 (4.2)	13 (18.1)	34 (47.2)	74 (18.6)
IIIa, n (%)	N/A	N/A	N/A	2 (18.2)	1 (9.1)	8 (72.7)	11 (2.8)
IIIb, n (%)	N/A	N/A	N/A	6 (15.8)	3 (7.9)	29 (76.3)	39 (9.8)
IVa, n (%)	1 (20.0)	3 (60.0)	N/A	N/A	1 (20.0)	N/A	5 (1.3)
IVb, n (%)	5 (41.7)	N/A	2 (16.7)	4 (33.3)	N/A	1 (8.3)	12 (3.0)
V, n (%)	N/A	N/A	N/A	N/A	N/A	N/A	13 (3.3)
Total, n (%)*	25 (6.3)	13 (3.3)	29 (7.4)	40 (10.2)	25 (6.3)	249 (63.2)	394 (24.2)

Complications graded by gravity and sub-characterizing according to organ system affected: cardiac, respiratory (resp.), neurological (neuro.), gastrointestinal (GI), renal and other as proposed in Dindo et al.^[16]^

GI = gastrointestinal.

* Deaths correspond to 13 (3.3%) of all complications and are not included in either organ system category.

The median postoperative length of hospital stay for patients admitted to the PACU during the study period was 2 days (IQR: 1–4 days). Additionally, 205 patients (12.5%) underwent reintervention within 30 days.

### 3.1. Risk factors

#### 3.1.1. Postoperative mortality rate (POMR)

POMR was higher among older patients, male patients, those with higher American Society of Anesthesiologists Physical Status (ASA-PS) scores, and patients with greater European Society of Anaesthesiology/European Society of Cardiology (ESA/ESC) surgical risk. Vascular surgery had a significantly higher POMR compared to all other surgical subtypes (3.4% vs 0.5%). There were no significant differences in POMR based on the type of anesthesia or the day of the week of the procedure (Table 3).

**Table 3:** Univariate analysis for POMR and major complications.

		Perioperative death		*P*-value	Major complications		*P*-value
		Dead, n = 13 (0.8)	Alive, n = 1629		Present, n = 80	Absent, n = 1562	
Age (yr)		79 (64–88)	58 (44–69)	<.001	65 (52–73)	58 (44–69)	<.001
Male, n (%)		11 (84.6)	770 (47.3)	.010	51 (63.7)	730 (46.7)	.004
ASA-PS, n (%)	I	0	176 (10.8)	<.001	3 (3.8)	173 (11.1)	<.001
	II	0	799 (49.0)		26 (32.5)	773 (49.5)	
	III	8 (61.5)	377 (23.1)		32 (40.0)	353 (22.6)	
	IV	3 (15.4)	21 (1.3)		7 (8.8)	17 (1.1)	
Procedure duration (min)		154 (84–210)	104 (65–165)	.087	171 (88–303)	103 (64–160)	<.001
SIGIC, n (%)		2 (15.4)	572 (35.1)	.241	12 (15.0)	562 (36.0)	<.001
Surgical specialty, n (%)	Gastrointestinal	2 (15.4)	130 (8.0)	.007	11 (13.8)	121 (7.7)	<.001
	Hepato-biliar	2 (15.4)	123 (7.6)		9 (11.3)	116 (7.4)	
	General – other subspeciality	1 (7.7)	306 (18.8)		6 (7.5)	301 (19.3)	
	Orthopedics	0	393 (24.1)		7 (8.8)	386 (24.7)	
	Ear, nose, mouth, and throat	1 (7.7)	86 (5.3)		2 (2.5)	85 (5.4)	
	Plastic surgery	1 (7.7)	142 (8.7)		13 (16.3)	130 (8.3)	
	Vascular	5 (38.5)	144 (8.8)		23 (28.7)	126 (8.1)	
	Urology	1 (7.7)	217 (13.3)		8 (10.0)	210 (13.4)	
	Others	N/A	88 (5.4)		1 (1.3)	87 (5.6)	
ESA risk, n (%)	Low	2 (15.4)	776 (47.6)	<.001	26 (32.5)	752 (48.1)	<.001
	Intermediate	6 (46.2)	778 (47.8)		30 (37.5)	754 (48.3)	
	High	5 (38.5)	75 (4.6)		24 (30.0)	56 (3.6)	
Anesthesia type, n (%)	General	8 (61.5)	782 (48.0)	.137	37 (46.3)	753 (48.2)	.556
	Locoregional	1 (7.7)	186 (11.4)		11 (13.8)	176 (11.3)	
	Combined	2 (15.4)	602 (37.0)		28 (35.0)	576 (36.9)	
	MAC	1 (7.7)	30 (1.8)		3 (3.8)	28 (1.8)	

Ear, nose, mouth, and throat – includes maxillofacial, stomatology and otorhinolaryngology; SIGIC; MAC. Missing data regarding ASA-PS for 258 (15.7%) patients, anesthesia type for 30 (1.8%) patients and procedure duration for 141 (8.6%) patients. Missing data was non-discriminant in POMR and major complication analysis. Mann–Whitney *U* test was applied for continuous variables (non-normal distribution across groups) and chi-square or Fisher’s exact test for categorical variables, as appropriate. Missing data were not discriminatory for the variables.

ASA-PS = American Society of Anesthesiologists Physical Status, ESA = European Society of Anaesthesiology, MAC = monitored anesthesia care, POMR = perioperative mortality rate, SIGIC = Sistema Integrado de Gestão de Inscritos para Cirurgia (Integrated Management System of the Waiting List for Surgery).

#### 3.1.2. Major complications

The incidence of major complications was higher among older patients, male patients, those with higher ASA-PS scores, and patients with greater ESA/ESC surgical risk. Longer procedure durations were also associated with more major complications. Vascular surgery patients experienced significantly more major complications than patients in other surgical groups (15.5% vs 3.8%; *P* < .001). Additionally, patients who underwent surgery on weekends (SIGIC) experienced fewer major complications compared to those who had surgery on weekdays (2.1% vs 6.4%; *P* < .001). No differences were found based on the type of anesthesia (Table 3).

#### 3.1.3. Weekday versus weekend surgery

Table S1 (Supplemental Digital File 3, Supplemental Digital Content, https://links.lww.com/MD/Q617) compares patient and procedural characteristics between elective surgeries performed on weekdays and those conducted on weekends under the SIGIC system.

Patients undergoing weekend procedures were slightly younger (median 57 years vs 58 years), but this difference was not statistically significant. The proportion of male patients was also similar between groups (45.6% vs 48.6%). Distribution across ASA-PS classes did not differ significantly.

Marked differences emerged in surgical duration and specialty distribution. Weekend procedures were significantly shorter, with a median duration of 77 minutes (IQR 46–110) compared to 128 minutes (IQR 84–191) on weekdays (*P* < .001). Gastrointestinal and vascular surgeries were proportionally more frequent during weekends (*P* < .001), whereas other subspecialty and orthopedic surgeries predominated during weekdays.

Regarding anesthesia, weekend procedures more often involved general anesthesia (53.5% vs 46.6%), while weekday surgeries more commonly used combined anesthesia techniques (40.9% vs 31.0%; *P* < .001).

Postoperative hospital stay was significantly shorter for patients undergoing weekend procedures, with a median of 1 day (IQR 1–3) versus 2 days (IQR 1–4) for weekday cases (*P* = .017).

#### 3.1.4. Multivariate analysis

Logistic regression was carried out with POMR as the dependent variable and age, sex, ASA-PS, procedure duration, surgical specialty, and ESA/ESC risk as covariates.

In the pooled model with imputed data, which included all 1642 patients, both age and higher ESA/ESC risk (vs low ESA/ESC risk) remained significantly related to POMR (*P* < .001 and 0.042, respectively), with an OR of 2.66 (95% CI: 1.37–5.19) for each 10-year increase and an OR of 13.94 (95% CI: 1.11–175.66) for higher ESA/ESC risk vs low ESA/ESC risk.

**Figure 2.**
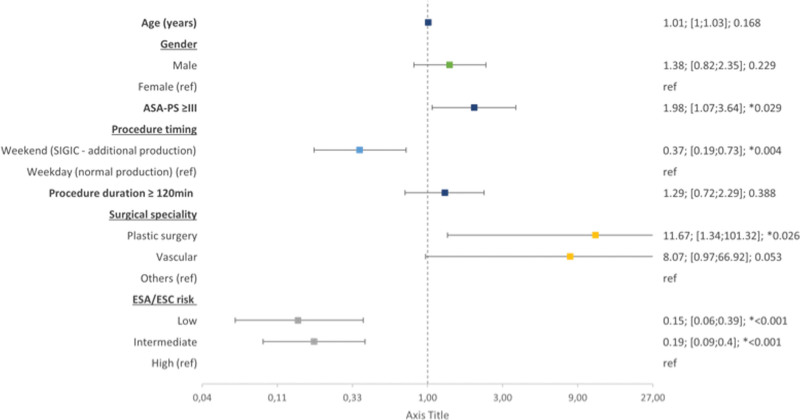
Multivariate analysis for major complications. Forest plot of the logistic regression with major complications as the dependent variable and age, sex, ASA-PS, procedure duration, SIGIC, surgical specialty, and ESA/ESC risk as covariates. OR; 95% CI; (ref) category; and * statistically significant at α = 0.05. The center of each square represents the OR for each considered variable, and the corresponding horizontal line represents the 95% confidence interval. ASA-PS = American Society of Anesthesiologists Physical Status, CI = confidence interval, ESA/ESC = European Society of Anaesthesiology/European Society of Cardiology, OR = odds ratio, ref = reference, SIGIC = Sistema Integrado de Gestão de Inscritos para Cirurgia (Integrated Management System of the Waiting List for Surgery).

Table 4 and Figure 2 show a logistic regression with major complications as the dependent variable and age, sex, ASA-PS, procedure duration, SIGIC, surgical specialty, and ESA/ESC risk as covariates. Similar results were found in this model: a higher ASA-PS (*P* = .029), greater ESA/ESC surgical risk (*P* < .001), non-SIGIC procedure (*P* = .004), and plastic procedure (*P* = .026) remained significantly associated with major complications, and vascular procedures, compared to all other procedures, remained marginally associated with major complications (*P* = .053).

**Table 4:** Multivariate analysis for major complications.

		Multiple imputation pooled model (n = 1642)	
		OR (95% CI)	*P*-value
Age (yr)		1.01 (1.00–1.03)	.168
Gender	Male	1.38 (0.82–2.35)	.229
	Female (ref)		
ASA-PS ≥ III		1.98 (1.07–3.64)	**.029**
Procedure timing	Weekend (SIGIC)	0.37 (0.19–0.73)	**.004**
	Weekdays (ref)		
Procedure duration ≥120 min		1.29 (0.72–2.29)	.388
Surgical speciality	Plastic surgery	11.67 (1.34–101.32)	**.026**
	Vascular	8.07 (0.97–66.92)	.053
	Others		
ESA/ESC risk	Low	0.15 (0.06–0.39)	**<.001**
	Intermediate	0.19 (0.09–0.40)	**<.001**
	High (ref)		

Logistic regression with major complications as the dependent variable, with age, sex, ASA-PS, procedure duration, SIGIC, surgical speciality, and ESA/ESC risk as covariates. ASA-PS was recategorized to ASA-PS ≥ III, procedure duration was recategorized in ≥120 min, surgical specialities were transformed into dummy variables, and then a new surgical specialty variable was created individualizing the specialties that were independently associated with complications versus all others, and ESA/ESC high risk group was considered as reference. A multiple imputation was applied to account for missing data, incorporating pooled estimates from 10 imputations based on a fully conditional specification imputation method with 10 iterations – pooled model is presented.

The bold values indicate statistically significant *P* < .05.

ASA-PS = American Society of Anesthesiologists Physical Status, CI = confidence interval, ESA = European Society of Anaesthesiology, ESC =European Society of Cardiology, OR = odds ratio, SIGIC = Sistema Integrado de Gestão de Inscritos para Cirurgia (Integrated Management System of the Waiting List for Surgery).

## 4. Discussion

This large monocentric study uniquely focuses on perioperative mortality and major morbidity of elective surgical patients during a pandemic wave, specifically examining the impact of the COVID-19 surge on elective surgeries at a large Portuguese academic medical center. We determined a POMR of 0.7% and a 30-day postoperative incidence of complications of 24.2%, with 13 deaths at 30 days. Older patients, men, and those with higher risk scores were more likely to experience severe complications after surgery. These complications were also more common with longer surgeries. Interestingly, weekend surgeries seemed to have fewer complications than weekday surgeries.

The POMR serves as an indicator of both surgical safety and access; limited access to surgery can result in delayed presentations and worse outcomes. The COVID-19 pandemic significantly impacted the Portuguese health system in 2020, restricting timely procedures and potentially leading to worse outcomes due to the advanced stages of diseases.^[3,4,24]^ Despite these challenges, our study found a relatively low POMR compared to the approximately 3% rates reported in Europe, the USA, and Brazil.^[25,26]^ This lower rate may be attributed to our focus on patients admitted to the PACU after elective noncardiac surgeries, as the admssion of all these elective surgical patients to PACU allowed the necessary elective surgeries to proceed while ICUs were overwhelmed by COVID 19 patients. Our findings are consistent with the GlobalSurg study,^[19]^ which reported a 1.4% POMR for cancer surgeries in high-income countries, emphasizing the role of postoperative care in reducing mortality.

SIGIC procedures were linked to lower odds of major complications. These findings contradict the “weekend effect,” which associates weekend healthcare with poor outcomes.^[9,27]^ This discrepancy may be due to our institution’s use of the SIGIC system,^[8]^ which schedules weekend elective procedures to reduce waiting times. In our study, weekend procedures were linked to a 60% reduction in the risk of major complications. The payment for each procedure is indexed to each grupos de diagnóstico homogéneo (homogeneous diagnosis groups, Portuguese DRG equivalent; GDH)-attributed value, resulting in additional fees/procedure for the team and the hospital and often requiring greater accuracy of records and safety checklists, as well as enhanced team efficiency to improve turnover and financial income/procedure.^[8,9]^ A potential source of confounding by indication exists in the comparison between SIGIC and non-SIGIC procedures. It is plausible that, particularly during the acute phases of the pandemic, patient selection for SIGIC procedures might have preferentially favored lower surgical and clinical risk patients, or those with more flexible scheduling requirements, potentially leading to their scheduling on weekends or nonpeak hours to optimize OR utilization for more urgent or complex cases during regular working hours. However, our multivariable analyses, which revealed an association between SIGIC procedures and reduced complications, were adjusted for key indicators of surgical and clinical risk, including ASA Physical Status, ESA/ESC surgical risk and procedure time. While these adjustments help to attenuate potential confounding by indication, we acknowledge that these are not exhaustive measures of risk, and residual confounding due to unmeasured factors inherent to scheduling practices during a crisis cannot be entirely ruled out. This potential bias should be considered when interpreting the observed differences in outcomes between these 2 groups. Further research is needed to identify all factors responsible for the observed reduction in complications under SIGIC, which may also include aspects such as the experience of surgical teams with a lower resident/specialist ratio, in hospital preoperative stay and optimization, and the increased motivation linked to higher remuneration for SIGIC procedures.^[9,28]^ That said, the insights gained from the SIGIC system during the pandemic may offer valuable lessons for other healthcare systems. While the specific structure of SIGIC is unique to Portugal, the principles of centralized management, transparent waiting lists, and standardized outcome reporting could be adapted to various national and regional contexts. However, successful transferability would necessitate careful consideration of existing healthcare infrastructure, funding models, cultural factors, and the integration with local clinical practices.

Our study also indicates that disruptions caused by surges seem to have significant deterimental effects on surgical complications. The pandemic significantly overwhelmed healthcare systems globally, both during the acute phase and in the subsequent recovery period, driving these systems to a critical saturation point due to shortages in human resources.^[2,6,29–31]^ This has impacted operational efficiency and patient safety.^[3,4]^ In Portugal, an effort to address surgical waiting lists involved the implementation of elective surgeries during overtime hours.^[5]^ Despite the preexisting use of this strategy, a recent report from the Partnership for Health System Sustainability and Resilience suggests that these measures have not been sufficient to fully meet the healthcare demands.^[5]^

To this day, hospitals still face challenges similar to those experienced during the pandemic, including resource shortages and ongoing backlogs of delayed elective surgeries.^[5,30,32]^ Health inequities persist, with vulnerable populations facing more significant barriers to accessing healthcare services.^[7]^ These ongoing issues highlight the need for continued healthcare infrastructure and support systems improvements. Despite these challenges, our study reports on a tertiary hospital that successfully resumed elective surgical activities during regular hours and overtime, achieving good clinical outcomes regarding postoperative complications and postoperative mortality rates.

Our study has several limitations. The POMR, defined by the WHO as the number of deaths during or after surgery divided by the number of procedures performed,^[11]^ varies across studies, affecting comparability and generalizability. We also encountered significant missing data for ASA-PS (15.7%), anesthesia type (1.8%), and procedure duration (8.6%), despite these being mandatory variables in surgical records. Besides the missing data for ASA-PS, anesthesia type, and procedure duration, there may be other unrecorded or inaccurately recorded variables that could impact the study’s results.

The study’s retrospective nature might introduce selection bias and limit the ability to control for all potential confounding factors. To mitigate this, we ensured a representative and comprehensive sample of all eligible elective surgeries performed during the study period. Surgery and clinical records were systematically analyzed by 2 researchers, thereby minimizing potential information bias. Despite these measures, we acknowledge that unmeasured confounders inherent to retrospective designs might still influence the observed associations. Since the study was conducted in a single academic medical center, the findings may not be generalizable to other institutions with different patient populations, resources, or practices. The unique circumstances of the COVID-19 pandemic, including altered hospital workflows and resource allocation, may not reflect normal operating conditions, potentially affecting the interpretation of the study’s findings.

The study included only elective noncardiac surgeries, excluding emergency, urgent, and cardiac procedures, which may limit the applicability of the results. While the Clavien–Dindo classification is widely accepted, it may still introduce variability in how complications are categorized and reported. Differences in anesthesia techniques and their implementation might vary, and impact outcomes, yet the study did not find significant differences based on anesthesia type. The focus on 30-day outcomes means that longer-term complications and mortality, which can also be significant, were not assessed. The potential benefits of weekend surgeries noted in this study may differ from other settings or might change as circumstances evolve, such as hospital policies or staffing changes. Finally, the study did not account for the potential impact of ethnic and socioeconomic factors on surgical outcomes, which could influence the results.

Despite its limitations, our study offers valuable insights into perioperative mortality and major morbidity during a pandemic wave, highlighting the effectiveness of the SIGIC system in managing elective surgery wait times. The rigorous data collection and advanced statistical methods enhance the robustness of the findings, providing practical implications for improving crisis management strategies and patient safety. Additionally, the study benchmarks perioperative outcomes, contributing to continuous improvements in surgical care practices and preparedness for future disruptions.

## Author contributions

**Conceptualization:** Ana Lídia Rouxinol-Dias, Cristina Granja.

**Data curation:** Ana Lídia Rouxinol-Dias, Leonardo Ferreira, Patrícia Martins Lima.

**Formal analysis:** Cláudia Camila Dias.

**Funding acquisition:** Ana Lídia Rouxinol-Dias.

**Investigation:** Ana Lídia Rouxinol-Dias, Diana Rodrigues, Leonardo Ferreira, Patrícia Martins Lima, Patrícia Ramos.

**Methodology:** Ana Lídia Rouxinol-Dias, Diana Rodrigues, Leonardo Ferreira, Patrícia Martins Lima, Patrícia Ramos, Cláudia Camila Dias.

**Project administration:** Joana Berger-Estilita, Cristina Granja.

**Supervision:** Joana Berger-Estilita.

**Validation:** Diana Rodrigues, Joana Berger-Estilita.

**Writing** – **original draft:** Ana Lídia Rouxinol-Dias, Cristina Granja.

**Writing** – **review & editing:** Joana Berger-Estilita, Cristina Granja.

## Supplementary Material



## References

[1] Deryugina T Gruber J Sabety A. Natural Disasters and Elective Medical Services: How Big is the Bounce-Back? (July 2020). NBER Working Paper No. w27505. https://ssrn.com/abstract=3649869 - Its a monography.

[2] Peralta Santos A. Three perspectives on the impact of the COVID-19 pandemic in Portugal 2023.

[3] Collaborative CO. Elective surgery cancellations due to the COVID-19 pandemic: global predictive modelling to inform surgical recovery plans. Br J Surg. 2020;107:1440–9. 32395848 10.1002/bjs.11746 PMC7272903

[4] Stahel PF. How to risk-stratify elective surgery during the COVID-19 pandemic? Patient Saf Surg. 2020;14:8. 32288785 10.1186/s13037-020-00235-9 PMC7107008

[5] Oliveira MD Tavares AI Vieira A Pacheco M. Sustainability and resilience in the portuguese health system. Partnership Health Syst Sustain Resilience. London School of Economics and Political Science (LSE) Consulting. 2022. https://www.phssr.org - Its a Report

[6] Frio GS Russo LX de Albuquerque CP. The disruption of elective procedures due to COVID-19 in Brazil in 2020. Sci Rep. 2022;12:10942. 35768482 10.1038/s41598-022-13746-5 PMC9243075

[7] Lin JA Braun HJ Schwab ME Pierce L Sosa JA Wick EC. Pandemic recovery: persistent disparities in access to elective surgical procedures. Ann Surg. 2023;277:57–65. 33914483 10.1097/SLA.0000000000004848 PMC8542562

[8] ACSS. Sistema Integrado de Gestão de Inscritos para Cirurgia (SIGIC). In: System CAotPH, editor.: Central Administration of the Health System (ACSS); 2011.

[9] Cristóvão R Gomes P. Waiting time policies in the health sector. In: Siciliani L Borowitz M Moran V, editors. OECD Health Policy Studies. Paris: OECD Publishing; 2013. p. 237–62.

[10] Vandenbroucke JP von Elm E Altman DG; STROBE Initiative. Strengthening the reporting of observational studies in epidemiology (STROBE): explanation and elaboration. PLoS Med. 2007;4:e297. 17941715 10.1371/journal.pmed.0040297 PMC2020496

[11] Organization WH. WHO 2015 Global Reference List of 100 Core Health Indicators. 2015.

[12] INE. Estatísticas da Saúde: 2019. 2021.

[13] Organization WH. Global reference list of 100 core health indicators. World Health Organization; 2015.

[14] Meara JG Leather AJ Hagander L. Global Surgery 2030: evidence and solutions for achieving health, welfare, and economic development. Lancet. 2015;386:569–624. 25924834 10.1016/S0140-6736(15)60160-X

[15] Watters DA. The value of reporting perioperative mortality rates (POMR). World J Surg. 2021;45:50–2. 33025155 10.1007/s00268-020-05804-8

[16] Dindo D Demartines N Clavien PA. Classification of surgical complications: a new proposal with evaluation in a cohort of 6336 patients and results of a survey. Ann Surg. 2004;240:205–13. 15273542 10.1097/01.sla.0000133083.54934.ae PMC1360123

[17] Clavien PA Barkun J de Oliveira ML. The Clavien-Dindo classification of surgical complications: five-year experience. Ann Surg. 2009;250:187–96. 19638912 10.1097/SLA.0b013e3181b13ca2

[18] Bolliger M Kroehnert JA Molineus F Kandioler D Schindl M Riss P. Experiences with the standardized classification of surgical complications (Clavien-Dindo) in general surgery patients. Eur Surg. 2018;50:256–61. 30546385 10.1007/s10353-018-0551-z PMC6267508

[19] GlobalSurg C, National Institute for Health Research Global Health Research Unit on Global S. Global variation in postoperative mortality and complications after cancer surgery: a multicentre, prospective cohort study in 82 countries. Lancet. 2021;397:387–97. 33485461 10.1016/S0140-6736(21)00001-5 PMC7846817

[20] Surgery NGHRUG. Quality and outcomes in global cancer surgery: protocol for a multicentre, international, prospective cohort study (GlobalSurg 3). BMJ Open. 2019;9:e026646. 10.1136/bmjopen-2018-026646 PMC6538014 31129582

[21] Bujang MA Sa’at N Sidik T Joo LC. Sample size guidelines for logistic regression from observational studies with large population: emphasis on the accuracy between statistics and parameters based on real life clinical data. Malays J Med Sci. 2018;25:122–30. 30914854 10.21315/mjms2018.25.4.12 PMC6422534

[22] Peduzzi P Concato J Kemper E Holford TR Feinstein AR. A simulation study of the number of events per variable in logistic regression analysis. J Clin Epidemiol. 1996;49:1373–9. 8970487 10.1016/s0895-4356(96)00236-3

[23] Upshur REG Moineddin R Crighton EJ Mamdani M. Seasonality of service provision in hip and knee surgery: a possible contributor to waiting times? A time series analysis. BMC Health Serv Res. 2006;6:22. 16509992 10.1186/1472-6963-6-22 PMC1420281

[24] Collaborative CO. Delaying surgery for patients with a previous SARS-CoV-2 infection. Br J Surg. 2020;107:e601–e2. 32974904 10.1002/bjs.12050 PMC7537063

[25] International Surgical Outcomes Study g. Global patient outcomes after elective surgery: prospective cohort study in 27 low-, middle- and high-income countries. Br J Anaesth. 2016;117:601–9. 27799174 10.1093/bja/aew316 PMC5091334

[26] Stefani LC Gamermann PW Backof A. Perioperative mortality related to anesthesia within 48 h and up to 30 days following surgery: a retrospective cohort study of 11,562 anesthetic procedures. J Clin Anesth. 2018;49:79–86. 29909205 10.1016/j.jclinane.2018.06.025

[27] Galyfos G Sigala F Bazigos G Filis K. Weekend effect among patients undergoing elective vascular surgery. J Vasc Surg. 2019;70:2038–45. 31147130 10.1016/j.jvs.2019.03.020

[28] Boas Práticas de Registos Clínicos em Cirurgia. 2016.

[29] Di Costanzo C. Healthcare resource allocation and priority-setting. a European challenge. Eur J Health Law. 2020;27:93–114. 33652412 10.1163/15718093-12271448

[30] Mehta A Awuah WA Ng JC. Elective surgeries during and after the COVID-19 pandemic: case burden and physician shortage concerns. Ann Med Surg (Lond). 2022;81:104395. 35999832 10.1016/j.amsu.2022.104395 PMC9388274

[31] Yoshida T Chude-Sokei R Araji T Adra S. Impact of COVID-19 pandemic surge on surgical outcomes: a retrospective study. Am Surg. 2024;90:1224–33. 38215308 10.1177/00031348241227213

[32] Vasconcelos R. Workers needed: a needed assessment of the workforce in local public health services in Portugal. Eur J Public Health. 2023;33(Supplement_2):322.

