# Supplementary material for: Elective surgery during a global health crisis – point incidence of mortality and complications in a Portuguese hospital: A cohort study
Source: Medicine (Baltimore). 2025 Nov 7;104(45):e45758. doi: 10.1097/MD.0000000000045758 (PMC12599731; doi:10.1097/MD.0000000000045758)
Supplement: Supplementary file 1 [file medi-104-e45758-s001.pdf]

Parecer da Comissão de Ética do

Centro Hospitalar Universitário de São João / Faculdade de Medicina da Universidade do Porto

Título do Projeto: Caracterização da morbimortalidade da UCPA no 3º trimestre de 2020

Nome da Investigadora Principal: Dra. Ana Lídia Rouxinol Sampaio Dias, interna de formação específica em Anestesiologia no CHUSJ

Onde decorre o Estudo: No Serviço de Anestesiologia do CHUSJ. Apresentou declaração do Prof. Doutor Fernando Abelha.

Objetivos do Estudo:
Caracterizar e reportar a incidência de morbimortalidade pós-operatória a 30 dias da Unidade de Cuidados Pós-anestésicos (UCPA), durante o período do terceiro trimestre de 2020.

Conceção e Pertinência do estudo:
As complicações pós-operatórias representam um fator de insatisfação para o doente e apresentam um elevado impacto económico nos cuidados de saúde. O reporte de morbimortalidade, quer a nível interno, quer a nível de publicação, podem ser importantes estratégias para sinalização e implementação de projetos de melhoria de cuidados de saúde 3-5.
O presente estudo pretende caracterizar e reportar a incidência de morbimortalidade pós-operatória a 30 dias no CHUSJ e identificar fatores associados à mesma, por forma a permitir a criação de estratégias de melhoria de cuidados de saúde associados ao peri-operatório dos doentes admitidos no Bloco Operatório Central e UCPA, com o intuito de reduzir a morbimortalidade associada.
Amostra consecutiva de todos os doentes acompanhados na UCPA, após terem sido submetidos a procedimentos cirúrgicos no BOC do CHUSJ entre 1 de outubro e 31 de dezembro de 2020.
Estão definidas as variáveis a recolher.

Benefício/risco: Não aplicável

Confidencialidade dos dados:
Cada doente receberá um identificador único gerado automaticamente na folha de registos, permitindo a anonimização dos dados a serem analisados.
Apresentou um pedido de reutilização de registos clínicos para Investigação e Desenvolvimento ao RAI.

Respeito pela liberdade e autonomia do sujeito de ensaio:
Dispõe de uma adequada informação ao participante e de modelo de CI do CHUSJ.

Curriculum **da investigadora**: Adequado à investigação.

Data previsível da conclusão do estudo: abril de 2021

Conclusão: Proponho um parecer favorável à realização do estudo.

Porto, 19 de fevereiro de 2021

O Relator da CE, Doutor Pedro Brito

Pedro Brito

Ethics Committee Approval

São João University Hospital Center / Faculty of Medicine, University of Porto

Project Title: Characterization of the Morbidity and Mortality of the Post-Anesthesia Care Unit in the 3rd Quarter of 2020 (protocol title)

Principal Investigator Dr. Ana Lídia Rouxinol Sampaio Dias, Anesthesiology Resident at CHUSJ

Study Location: Department of Anesthesiology, CHUSJ. Presented the approval declaration from the director of the department - Prof. Dr. Fernando Abelha.

Study Objectives: To characterize and report the incidence of postoperative morbidity and mortality at 30 days in the Post-Anesthesia Care Unit (PACU) during the third quarter of 2020.

Study Design and Relevance: Postoperative complications are a source of patient dissatisfaction and have a significant economic impact on healthcare. Reporting morbidity and mortality, both internally and through publication, can be crucial strategies for identifying and implementing healthcare improvement projects. This study aims to characterize and report the incidence of 30-day postoperative morbidity and mortality at CHUSJ and identify associated factors, thereby enabling the development of strategies to improve perioperative care for patients admitted to the Surgery Center and PACU, with the goal of reducing associated morbidity and mortality. A consecutive sample of all patients monitored in the PACU after undergoing surgical procedures in the CHUSJ Surgery Center between October 1 and December 31, 2020, can be included. The variables to be collected have been defined.

Benefit/Risk: Not applicable.

Data Confidentiality: Each patient will receive a unique identifier automatically generated on the registration sheet, ensuring the anonymization of the data to be analyzed. A request for the reuse of clinical records for Research and Development was submitted to Access to Information Officer.

Respect for the Autonomy of the Trial Subject: Adequate information for participants and an informed consent model from CHUSJ are provided.

Investigator's Curriculum: Suitable for the investigation.

Expected Date of Study Completion: April 2021

Conclusion: I propose a favorable opinion for the conduct of the study.

Porto, February 19, 2021

Ethics Committee Rapporteur, Dr. Pedro Brito

Table S1 -– Comparison elective in-hospital weekday vs weekend surgery

		**Weekdays** n=13(0.8)	**Weekend (SIGIC)** n=1629	**p-value**
**Age (years), p50 (p25-p75)**		58 (43-70)	57 (45-68)	0.313
**Male, n(%)**		519 (48.6)	262 (45.6)	0.254
**ASA-PS n(%)**	I	107 (11.8)	69 (14.4)	0.237
	II	517 (57.1)	282 (58.9)	
	III	263 (29.1)	122 (25.5)	
	IV	18 (2.0)	6 (1.3)	
**Procedure duration (min), p50 (p25-p75)**		128 (84-191)	77 (46-110)	<0.001
**Surgical specialty, n(%)**	Gastro-intestinal	106 (9.9)	26 (4.5)	<0.001
	Hepato-biliar	67 (6.3)	58 (10.1)	
	General – other subspeciality	177 (16.6)	130 (22.6)	
	Orthopedics	268 (25.1)	125 (21.8)	
	Ear, Nose, Mouth & Throat	55 (5.1)	32 (5.6)	
	Plastic surgery	99 (9.3)	44 (7.7)	
	Vascular	**59 (5.5)**	**90 (15.7)**	
	Urology	155 (14.5)	63 (11.0)	
	Others	82 (7.7)	6(1.0)	
**ESA risk n(%)**	Low	508 (47.6)	270 (47.0)	
	Intermediate	494 (46.3)	590 (50.5)	
	High	66 (6.2)	14 (2.4)	
**Anesthesia type n(%)**	General	488 (46.6)	302 (53.5)	<0.001
	Locoregional	111 (10.6)	76 (13.5)	
	Combined	**429 (40.9)**	**175 (31.0)**	
	MAC	20 (1.9)	11 (2.0)	
**Post-surgical hospital stay (days), p50 (p25-p75)**		2 (1-4)	1 (1-3)	0.017

Missing data regarding ASA-PS for 258 (15.7%) patients, anesthesia type for 30 (1.8%) patients and procedure duration for 141 (8.6%) patients. Missing data was non-discriminant for ASA-PS for 258 (15.7%) patients and anesthesia type but not procedure time.
